# People with higher systemizing traits have wider right hands

**DOI:** 10.3389/fpsyt.2024.1404559

**Published:** 2024-09-05

**Authors:** Na Chen, Souta Hidaka, Naomi Ishii, Makoto Wada

**Affiliations:** ^1^ Developmental Disorders Section, Department of Rehabilitation for Brain Functions, Research Institute of National Rehabilitation Center for Persons with Disabilities, Tokorozawa, Saitama, Japan; ^2^ Department of Psychology, Rikkyo University, Niiza, Saitama, Japan; ^3^ Department of Psychology, Faculty of Human Sciences, Sophia University, Chiyoda, Tokyo, Japan; ^4^ Information and Support Center for Persons with Developmental Disorders, National Rehabilitation Center for Persons with Disabilities, Tokorozawa, Saitama, Japan

**Keywords:** hand configuration, systemizing quotient, finger length, width-to-length ratio, metacarpophalangeal joints

## Abstract

**Introduction:**

Various genetic mutations have been implicated in autism spectrum disorder (ASD). Some candidate genes for ASD are known to be related to signal transduction and may be involved in hand development as well as neurodevelopment. Therefore, although subtle, anatomical variations in hand configurations may be observed in individuals with ASD. However, except for research on the finger ratio, which has been suggested to be related to prenatal sex hormone exposure, only few studies have been conducted. Given the spectrum characteristics of ASD, we explored whether hand configurations are associated with ASD-related traits in the general population.

**Methods:**

Photographs of the dorsal surface of each hand were obtained, and the distances between the metacarpophalangeal joints and finger lengths were measured. The Autism Spectrum Quotient, Empathy Quotient, and Systemizing Quotient were used to evaluate ASD-related traits.

**Results:**

We found a significant positive correlation between the aspect ratio of the right hand and the Systemizing Quotient score: individuals with a larger width relative to the finger length showed more systemizing traits.

**Discussion:**

These findings suggest that gene polymorphisms or prenatal sex hormone exposure may underlie the relationship between systemizing traits and hand configurations.

## 1 Introduction

Autism spectrum disorder (ASD) is a neurodevelopmental disorder characterized by difficulties in social communication (difficulties in social interactions and communication) and restricted or repetitive patterns of behaviors, interests, and activities (1, 2). The prevalence of ASD has steadily increased over the past few decades, and it is estimated that 1 out of 54 children has been diagnosed with ASD in the United States (3). In Japan, the prevalence of ASD among preschool children is approximately 3% (4, 5).

Given the spectrum characteristics of ASD, it is likely that the various ASD-related traits are widely distributed in the general population (6–8), and these traits can be assessed using several questionnaires, as described below. Based on the “extreme male brain” theory, which posits that masculine brain characteristics are extreme in individuals with ASD (9–11), ASD-related traits can be framed using two dimensions in the neurotypical mind: systemizing and empathizing (12). Empathizing traits are defined as sensitivity to social cues and the tendency to have concomitant emotional reactions with others; females generally have higher Empathy Quotient (EQ) scores than males (13). Systemizing traits are related to the focus on detecting abstract rules that govern systems, such as specific classification characteristics or understanding number patterns; males generally have higher Systemizing Quotient (SQ) scores than females. Higher SQ scores and lower EQ scores are relatively common among typically developing males, with extreme cases being individuals with ASD (12, 14, 15).

Additionally, more than 1000 candidate genes have been identified, suggesting that various genes are involved in causing ASD (16, 17). Many of these genes are involved in neuronal development, including synaptic connectivity and gene expression regulation. Variations in these genes have been suggested to cause ASD by influencing the neurodevelopmental process. Among them, mutations in the *wnt* gene, which controls neurodevelopment via gene expression regulation, have been reported to cause ASD (18). The *wnt* gene is also known to be involved in hand development through the regulation of *Hox* gene cluster expression (19). Therefore, individuals with ASD may show characteristic shape changes in their fingers. In addition, gene polymorphisms in the neurexin gene have recently been reported to be associated with individual characteristics of systemizing traits, one of the ASD-related traits (20). This gene is also associated with the expression of bone morphogenetic proteins, which may influence hand development (21). Therefore, it is possible that gene polymorphisms widely influence hand development. Because minor physical anomalies, including hand anomalies, sometimes co-occur in various psychiatric diseases or neurodevelopmental disorders (22–25), subtle hand configurations unique to ASD-related traits may be observed. Hence, based on the findings of previous studies, we hypothesized that the characteristics of hand configurations may be associated, at least in part, with ASD-related traits.

To date, several studies have focused on the relationship between ASD-related traits and the ratio of the length of the second finger (index finger) to the length of the fourth finger (ring finger) (2D:4D ratio) (26–31). For instance, Hönekopp (27) showed that the digit ratio (the 2D:4D ratio) was, on average, lower among individuals with clinically diagnosed ASD. Additionally, several studies have investigated the relationship between the 2D:4D ratio and personality traits, including ASD-related traits (Autism Spectrum Quotient [AQ], SQ, and EQ) in some populations (26, 28, 32, 33). Exposure to prenatal sex hormones, specifically testosterone, has been suggested to be related to the 2D:4D ratio (34). Males generally have a lower 2D:4D ratio than females, which corresponds to more male-typical characteristics (35). According to a previous study, male fetuses are exposed to at least 2.5 times higher testosterone levels than female fetuses between 8 and 24 weeks of gestation (36). Prenatal testosterone exposure has been suggested to increase systemizing traits and decrease empathizing traits in typically developing males and to possibly result in ASD via brain hypermasculinization (12, 14, 27, 37–41). Nevertheless, the utility of the 2D:4D ratio as an index of prenatal sex hormone exposure remains controversial (42). Furthermore, some studies suggest that the relationship between ASD-related traits (i.e., AQ, SQ, and EQ) and the digit ratio is not always significant (27, 43).

Conversely, except for research on the 2D:4D ratio, few studies have investigated the relationship between hand configurations and ASD-related traits. In particular, only a few anatomical studies have assessed the relationship between the hand width (i.e., the width between adjoining fingers [metacarpophalangeal (MCP) joints]) and the occurrence of carpal tunnel syndrome (orthopedic disease) or sexual dimorphism (44, 45). Sanfilippo et al. (45) examined the distances between various locations on the hand and used principal component analysis and so on to analyze sex differences in the anatomical features of the hand. Neither study targeted the relationship with ASD-related traits. To our knowledge, no study has examined the possible relationship between hand configurations, other than the 2D:4D ratio, and ASD-related traits, which may be influenced by gene polymorphisms or early prenatal sex hormone exposure. Assuming commonality in the genes involved in hand development and neurodevelopment, we considered the possibility that there are anatomical features associated with ASD-related traits.

This study aimed to explore the relationship between hand configurations, including the aspect ratio of the hand, and ASD-related traits (i.e., AQ, SQ, and EQ) in the general population. In this study, hand width was measured as the distance between the adjoining MCP joints of the fingers, and finger length was measured as the distance from the MCP joint to the tip of the third finger (i.e., the middle finger, which is the longest finger). The aspect ratio of the hand was calculated as the ratio of the hand width to the vertical finger length of the third finger. Given that the distance between the MCP joints is affected by hand size, with males generally having larger hands (46, 47), calculating the aspect ratio controls for the artificial effects of hand size. Subclinical ASD characteristics were evaluated using the AQ (6), SQ (10), and EQ (13). As an exploratory study, a correlation analysis between the aspect ratios of hands and AQ, SQ, and EQ scores was conducted in the general population.

## 2 Materials and methods

### 2.1 Participants

A total of 82 neurotypical participants (48 females, 34 males; mean age: 25.3 ± 8.72 years, ranging from 16 to 58 years), recruited from universities and other institutions near our institute, were included in this study. The laterality quotient (LQ) of each participant was estimated according to the Japanese version of the FLANDERS handedness questionnaire (48), and all participants were classified as right-hand dominant. Inclusion criteria for participants were as follows: (a) age 16–60 years; (b) right-handed (LQ ≥ 60); and (c) no history of current psychiatric or neurological disorders, including ASD. A prior power analysis using G*Power version 3.1 (49, 50) determined that a sample of 82 individuals would be sufficient to detect a correlation coefficient of 0.3, with an alpha of 0.05 and a power of 80% (51). This study was approved by the ethics committee of the National Rehabilitation Center for Persons with Disabilities (approval no. 2020-091) and was conducted in accordance with the relevant regulations and guidelines of the Ministry of Health, Labor, and Welfare of Japan. Written informed consent was obtained in advance from all participants.

### 2.2 Measurements and procedures

**Figure 1:**
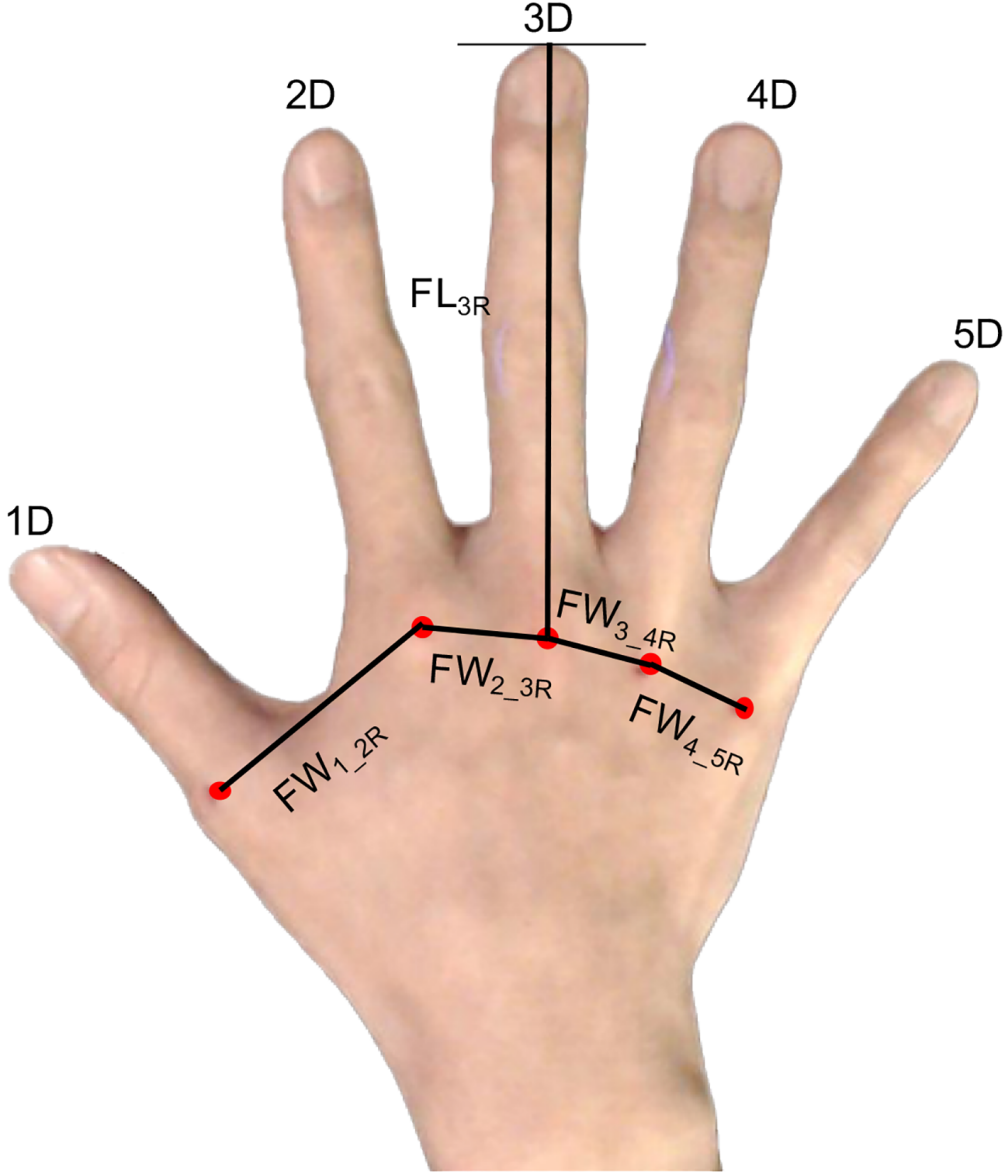
Points of the hand used to measure the distance between the points. FL3R, finger length of the third finger from the metacarpophalangeal (MCP) joints to the fingertip; FW1_2R, distance between the MCP joints of the thumb and second finger of the right hand; FW2_3R, distance between the MCP joints of the second finger and third finger; FW3_4R, distance between the MCP joints of the third finger and fourth finger joints; FW4_5R, distance between the MCP joints of the fourth finger and fifth finger.

The hands of the participants were individually photographed, and the participants completed the Japanese versions of the Autism Spectrum Quotient (AQ) (52), Empathizing Quotient (EQ), and Systemizing Quotient (SQ) (53), which are self-reported questionnaires. Prior to the photography session, the participants were instructed to clench their fists to reveal the MCP joints on the dorsum of both hands. Subsequently, an experimenter marked the MCP joints of the right and left hands with red using a cosmetic lip liner. The participants were guided to position their hands so that two photographs of the dorsal surface of both hands could be taken. They were asked to place their hands on a paper board (45 cm × 30 cm) with a 1-cm grid, positioned against a black background on a desk directly beneath a downward-facing web camera. The web camera (Logicool HD Pro C920r; Logitech International S.A., Lausanne, Switzerland) was mounted on a ball head (Velbon QHD-63; Hakuba Photo Industry Co., Ltd., Tokyo, Japan) and used to take photographs from a height of approximately 53 cm directly above the plate on the desk. The participants were instructed to naturally place their hands on the board, and photographs of the dorsal surface of their right and left hands were obtained. Photography was controlled using MATLAB (R2019b; MathWorks, Inc., Natick, MA, USA) on a computer (MAC mini, Apple, Inc., Cupertino, CA, USA). The distances between the MCP joints and the finger lengths were measured on the acquired images, and the actual dimensions were calculated from the grid (1 cm). The points on the hand and the measurements of the right hand are shown in **Figure 1**.

### 2.3 Autism spectrum quotient, empathizing quotient, and systemizing quotient

**Figure 2:**
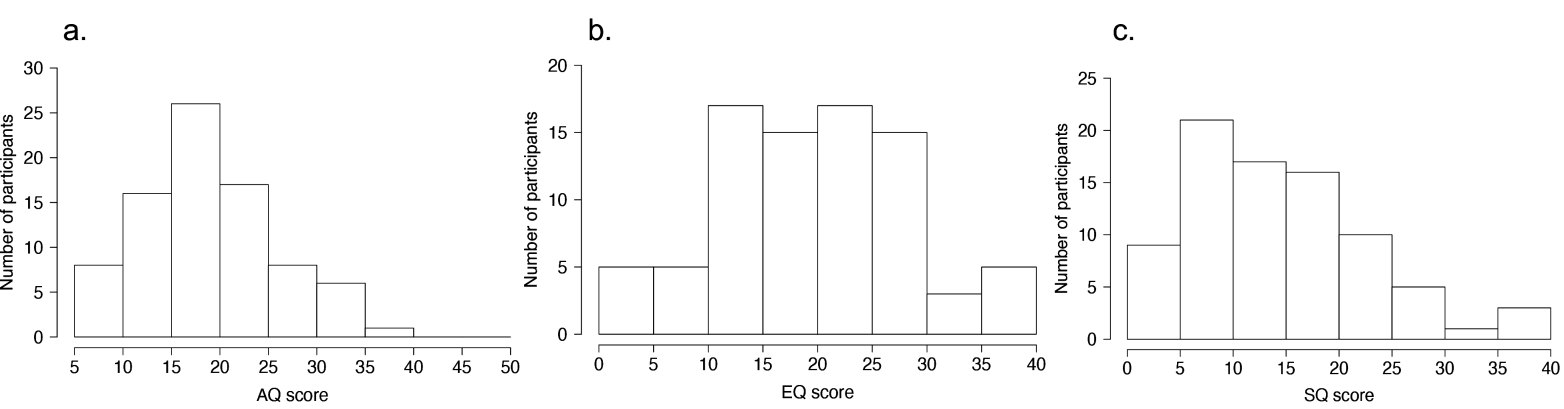
Distributions of ASD-related traits. **(A)** Distribution of Autism Spectrum Quotient (AQ) scores. **(B)** Distribution of Empathy Quotient (EQ) scores. **(C)** Distribution of Systemizing Quotient (SQ) scores.

The AQ is a self-reported questionnaire used for assessing autistic traits (6). The participants were instructed to rate the degree to which each item applied to them using a 4-point scale (1 point, definitely agree; 2 points, slightly agree; 3 points, slightly disagree; 4 points, definitely disagree). For example, item 1: “I prefer to do things with others rather than on my own.” Cronbach’s alpha value of the Japanese version was 0.81 (52). The AQ scores were calculated based on previous studies (6, 52). Higher AQ scores indicated a greater magnitude of ASD-related traits. The distributions of AQ scores (mean: 19.5 ± 6.84) are shown in **Figure 2A**.

The Japanese short versions of the EQ and SQ scores (53), based on the original version (54), were also used. The EQ and SQ scores measure the interests of individuals and their traits to empathize (taking on the perspectives of others and knowing what others are thinking) and systemize (understanding processes, systems, and machines, and identifying patterns, rules, or laws). Cronbach’s alpha values of the Japanese version of EQ and SQ scores were 0.86 and 0.89, respectively (53). The EQ and SQ scores were calculated based on a previous study (53, 54). Higher EQ and SQ scores indicated higher empathizing and systemizing traits, respectively. The distributions of EQ scores (mean: 19.9 ± 8.82) and SQ scores (mean: 14.7 ± 8.64) are shown in **Figures 2B, C**, respectively.

### 2.4 Data analysis

The AQ, EQ, and SQ scores and the measurements of hand configurations for each hand (**Table 1**) did not significantly deviate from the normal distribution (all *p* >.05, Shapiro–Wilk test). Because the purpose of this study was to examine the relationship between hand configuration and ASD-related traits, the width between the adjoining MCP joints was used as the main measurement (**Table 1**). To prevent the effect of hand size, the ratio of the distance between the MCP joints (*FW_2_3R/L_*, *FW_3_4R/L_*, and *FW_4_5R/L_*) to the length of the middle finger (*FL_3R/L_*), which was the longest part in the hand, was calculated, and logarithmic values were used for the correlation analysis. For example, the aspect ratio of the right hand was calculated as follows (Equation 1):

**Table 1:** Means and standard deviations of hand configuration measurements (cm) (*N* = 82).

	FW_1_2_	FW_2_3_	FW_3_4_	FW_4_5_	FL_1_	FL_2_	FL_3_	FL_4_	FL_5_
Right hand	4.62 (0.54)	2.16 (0.20)	1.87 (0.17)	1.83 (0.16)	5.79 (0.38)	9.00 (0.51)	10.07 (0.56)	9.42 (0.54)	7.31 (0.45)
Left hand	4.83 (0.58)	2.08 (0.20)	1.84 (0.16)	1.75 (0.15)	5.96 (0.42)	9.15 (0.56)	10.11 (0.59)	9.43 (0.58)	7.24 (0.48)

Means and standard deviations of hand configuration measurements (cm) (N = 82). FL3, finger length of the third finger from the metacarpophalangeal (MCP) joints to the fingertip; FW1_2, distance between the MCP joints of the thumb and second finger; FW2_3, distance between the MCP joints of the second finger and third finger; FW3_4, distance between the MCP joints of the third finger and fourth finger joints; FW4_5, distance between the MCP joints of the fourth finger and fifth finger.


(1) $$\log_{10}\frac{\left(FW_{2\_3R}+FW_{3\_4R}+FW_{4\_5R}\right)}{FL_{3R}}$$

Previous studies reported that AQ, EQ, and SQ scores were interrelated (55, 56). We found that the aspect ratio of the right hand (**Supplementary Table 1**) and some of the ratios (e.g., *FW_2_3R_*) showed a correlation with age; however, they were not correlated with handedness (**Supplementary Tables 1**, **2**). Hence, partial correlations using the AQ, EQ, or SQ scores and age were employed for the correlation analysis between the hand configuration ratios and ASD-related traits, with Pearson’s method applied. For example, when calculating the partial correlation between the aspect ratio and SQ score, we included AQ, EQ, and age as control variables. Bonferroni correction for multiple testing was applied to adjust the *p*-values.

We also evaluated sex differences in ASD-related traits (AQ, EQ, and SQ), LQ, and the ratios of hand configurations (**Supplementary Table 3**). We found significant sex differences in SQ and some ratios for hand configurations. To assess possible effects of sex differences, we performed hierarchical multiple linear regression analyses for the aspect ratio and the other ratios for hand configurations using the forced entry method with sex as a variable. Initially, sex was included as an explanatory variable in a model. Subsequently, we entered age, AQ, EQ, and SQ scores as explanatory variables into the model and tested whether the regression significantly increased. This analysis enabled us to assess the possible effects of sex differences while controlling for covariate relationships among AQ, EQ, and SQ scores, as in the partial correlation analyses.

As reference information, we calculated the 2D:4D ratio for each hand using photographs of the palm side, as described in previous studies (57). For the right hand, the sample size was N = 81 due to missing data. A partial correlation analysis of the 2D:4D ratio and AQ, SQ, and EQ scores was conducted to enable comparisons with previous studies.

Data analyses were performed using R software version 4.0.2 (R Core Team, https://www.R‐project.org/) and JASP 0.18.3 (JASP Team, https://jasp-stats.org/).

## 3 Results

### 3.1 Hand measurements

The means and standard deviations of each measurement of the right and left hands are presented in **Table 1**. We examined whether correlations exist between the hand configuration (i.e., the ratio of the finger width to the finger length) of each hand and ASD-related traits (**Figure 1**). To prevent the influence of hand size, the ratio of the distance between the MCP joints (*FW_2_3R/L_*, *FW_3_4R/L_*, and *FW_4_5R/L_*) to the length of the middle finger (*FL_3R/L_*) was calculated, and logarithmic values were used for the correlation analysis of ASD-related traits.

### 3.2 Relationships between the aspect ratio of the hand and ASD-related traits

As described in the Materials and Methods section, some of the metrics were correlated with age (**Supplementary Table 1**). Additionally, previous studies reported that the AQ, EQ, and SQ scores were interrelated (55, 56). Hence, partial correlations based on the AQ, EQ, or SQ scores and age were used for the correlation analysis, performed using Pearson’s method.

**Figure 3:**
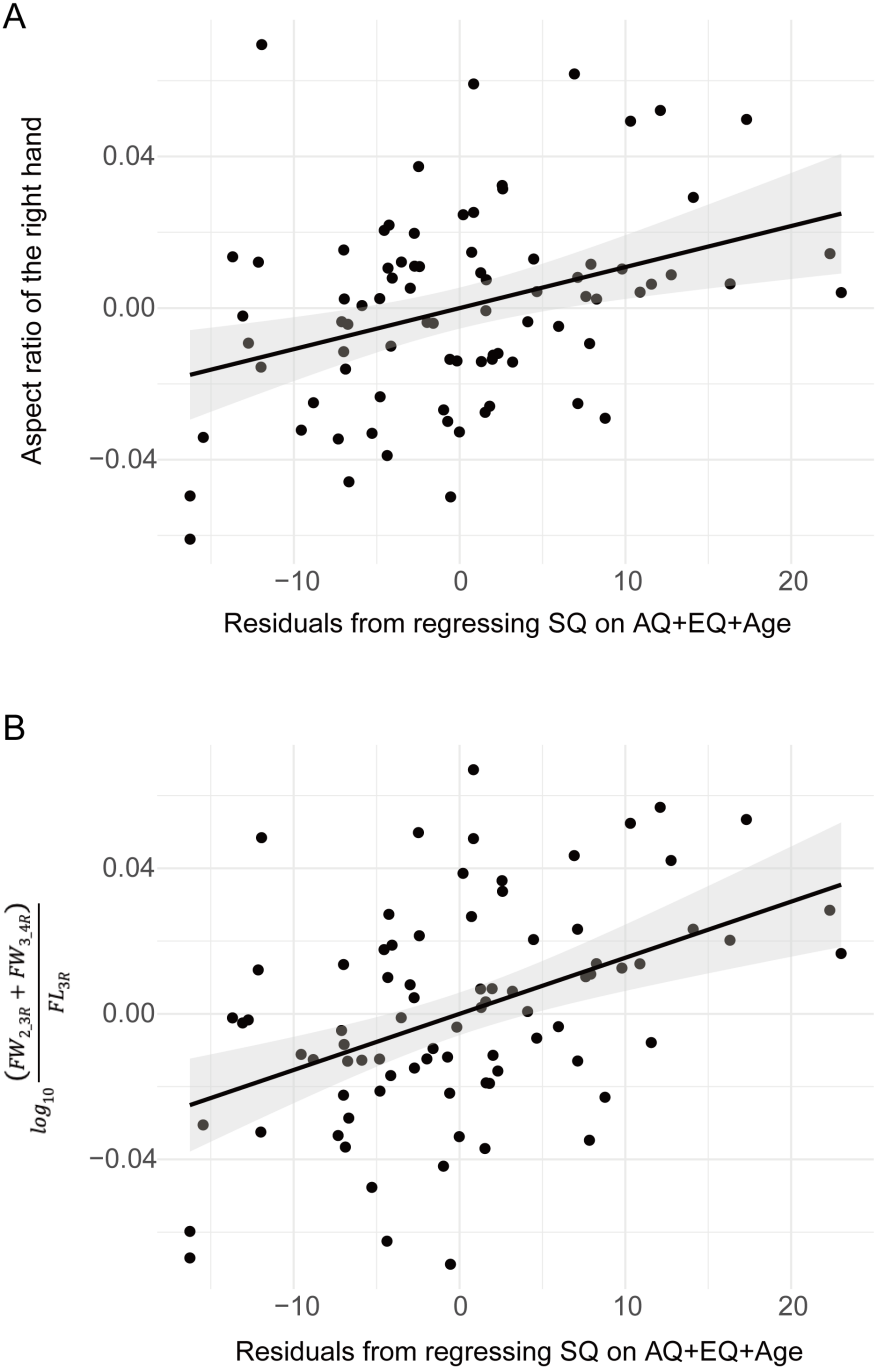
Relationship between the width-to-length ratio of the right hand and SQ scores. **(A)** Partial correlation between Systemizing Quotient (SQ) scores and the aspect ratio of the right hand (Equation 1) based on Autism Spectrum Quotient (AQ) + Empathy Quotient (EQ) + age. **(B)** Partial correlation between SQ scores and Log ((FW_2_3R_ + FW_3_4R_)/FL_3R_) based on AQ + EQ + age. FL_3R_, finger length of the third finger from the metacarpophalangeal (MCP) joints to the fingertip of the right hand; FW_2_3R_, distance between the MCP joints of the second finger and third finger of the right hand; FW_3_4R_, distance between the MCP joints of the third finger and fourth finger joints of the right hand.

With respect to the aspect ratio of the hand, we calculated the ratio of the distance between the MCP joints of the index and fifth fingers to the length of the middle finger (i.e., the ratio of the width of the hand to the length of the middle finger; log ((*FW_2_3R_* + *FW_3_4R_* + *FW_4_5R_*)*/FL_3R_*, Equation 1). The aspect ratio of the right hand was significantly correlated with the SQ scores (*r* = 0.35, *p* = .0016 <.05/3, Bonferroni correction) (**Figure 3A**) but was not significantly correlated with the AQ scores (*r* = –0.21, *p* = .067) and EQ scores (*r* = –0.047, *p* = .68) (**Table 2**). Additionally, the aspect ratio of the left hand was not significantly correlated with the AQ, SQ, and EQ scores (*r* = 0.090, *p* = .43; *r* = –0.091, *p* = .43; and *r* = –0.11, *p* = .33, respectively) (**Table 2**). We also evaluated sex differences in ASD-related traits (AQ, EQ, and SQ), LQ, and the ratios of hand configurations (**Supplementary Table 3**). Significant sex differences in the aspect ratio of the hand were not observed for either the right and left hand (*t*_80_ = −1.32, *p* = 0.19, Cohen’s *d* = –0.30; *t*_80_ = 0.007, *p* = 0.99, Cohen’s *d* = 0.002) (**Supplementary Table 3**).

**Table 2:** Partial correlation coefficients between hand configurations (log-transformed) and autistic traits.

		Right hand			Left hand		
		FW_2_3_/FL_3_	FW_3_4_/FL_3_	FW_4_5_/FL_3_	FW_2_3_/FL_3_	FW_3_4_/FL_3_	FW_4_5_/FL_3_
AQ	*r* = *p =*	–0.058 .61	–0.12 .30	0.022 .85	0.11 .34	0.0046 .97	–0.076 .51
SQ	*r* = *p =*	**0.35** ^*^ **.0013**	**0.36** ^*^ **.0012**	0.013 .91	0.088 .44	0.070 .54	0.1 .29
EQ	*r* = *p =*	–0.034 .77	–0.085 .46	0.024 .83	0.025 .83	0.14 .21	–0.056 .63
Age	*r* = *p =*	**0.32** ^*^ .0036	0.11 .34	0.19 .098	0.38^*^ **.00062**	0.32^*^ **.0046**	0.10 .37

Partial correlation coefficients between hand configurations (log-transformed) and autistic traits. FL3, finger length of the third finger from the metacarpophalangeal (MCP) joints to the fingertip; FW1_2, distance between the MCP joints of the thumb and second finger; FW2_3, distance between the MCP joints of the second finger and third finger; FW3_4, distance between the MCP joints of the third finger and fourth finger joints; FW4_5, distance between the MCP joints of the fourth finger and fifth finger. AQ, Autism Spectrum Quotient; EQ, Empathy Quotient; SQ, Systemizing Quotient. Values displayed in bold and asterisks indicate the level of statistical significance (*p < 0.05/12).

Since there was a significant difference in sex for the SQ score, we conducted hierarchical multiple linear regression analyses for the aspect ratio. Initially, sex was included as an explanatory variable in the model. Next, we added age, AQ, EQ, and SQ scores as explanatory variables into the model. In the first step, we did not find a significant model fit in the aspect ratio of the right hand (log ((*FW_2_3R_* + *FW_3_4R_* + *FW_4_5R_*)*/FL_3R_*): N = 82, *F*(1, 80) = 1.75, *p* = .19, adjusted *R* ^2 ^= 0.009). In the second step, adding the SQ score and age resulted in a significant change (*t* = 2.35, *p* = .021; *t* = 2.91, *p* = .005) in the explanatory effect of the model (*F*(5, 76) = 3.95, *p* = .003, adjusted *R* ^2 ^= 0.154, *R* ^2^ changes = 0.185, **Supplementary Table 4A**). The main findings are consistent with those of the aforementioned analyses. With regard to the left hand, adding age, AQ, EQ, and SQ scores did not result in significant *R* ^2^ changes in the explanatory effect of the model (**Supplementary Table 4B**).

### 3.3 Relationships between other hand configurations and ASD-related traits

Similar analyses were performed for the distance between each of the MCP joints to determine which sites were associated with systemizing traits. The partial correlation analysis showed that the ratio of several distances between the MCP joints to the length of the middle finger of the right hand was significantly correlated with SQ scores (log (*FW_2_3R_/FL_3R_*): *r* = 0.35, *p* = .0013; log (*FW_3_4R_/FL_3R_*): *r* = 0.36, *p* = .0012) (**Table 2**). Furthermore, the ratio of the distance between the MCP joints of the ring and little fingers (*FW_4_5R_*) to the length of the middle finger (*FL_3R_*) exhibited no significant correlation with SQ scores (log (*FW_4_5R_/FL_3R_*): *r* = 0.013, *p* = .91) (**Table 2**). The ratios were not significantly correlated with the AQ and EQ scores (**Table 2**). The ratios of the hand configuration of the left hand were not significantly correlated with the AQ, SQ, and EQ scores (**Table 2**). The width between the index-finger and ring-finger MCP joints adjusted by the length of the middle finger contributed to the significant correlation between the hand configuration of the right hand and SQ scores. A significant partial correlation was observed between the ratio of the distance from the index-finger to the ring-finger MCP joints (*FW_2_3R_* + *FW_3_4R_*) and the length of the middle finger (*FL_3R_*) of the right hand (log ((*FW_2_3R_* + *FW_3_4R_*)*/FL_3R_*)): *r* = 0.44, *p* <.0001) (**Figure 3B**).

Significant differences were observed in sex for some ratios (**Supplementary Table 3**). The hierarchical multiple linear regression analyses, with sex as a variable, found no significant model fit for the ratio of the distance between the MCP joints of the index and middle fingers (*FW_2_3R_*) to the length of the middle finger (*FL_3R_*) of the right hand (log (*FW_2_3R_/FL_3R_*): N = 82, *F*(1, 80) = 0.10, *p* = .75, adjusted R^2^ = -0.011) in the first step. In the second step, the inclusion of SQ score and age induced a significant change (*t* = 3.09, *p* = .003; *t* = 2.60, *p* = .011) in the explanatory effect of the model (*F*(5, 76) = 4.13, *p* = .002, adjusted *R* ^2^ = 0.21, *R* ^2^ changes = 0.21, **Supplementary Table 5A**). The main findings are consistent with those obtained from the aforementioned analyses. With regard to the other ratios (log (*FW_3_4R_/FL_3R_*), log (*FW_4_5R_/FL_3R_*), log (*FW_2_3L_/FL_3L_*), log (*FW_3_4L_/FL_3L_*), log (*FW_4_5L_/FL_3L_*)), input of age, AQ, EQ and SQ scores did not induce significant *R* ^2^ changes in the explanatory effect of the model (**Supplementary Tables 5B–F**).

### 3.4 Relationships between the 2D:4D ratio and ASD-related traits

As a reference information, we calculated the 2D:4D ratio of each hand and examined its correlation with AQ, SQ, and EQ scores by conducting a partial correlation analysis to enable comparisons with previous studies (26, 27, 32). The results indicated no significant correlation between the 2D:4D ratio of each hand and the SQ scores (**Supplementary Table 6**). Regarding the aspect ratios, no significant correlation was found between the aspect ratio and the 2D:4D ratio of the right hand, while a significant correlation was found for the left hand (**Supplementary Table 7**).

## 4 Discussion

This study revealed a significant correlation between the aspect ratio of the right hand (i.e., the ratio of the width of the finger MCP joints to the finger length) and systemizing traits in the general population. We observed that participants with a wider hand width (e.g., distance of the MCP joints from the index finger to the ring finger) tended to have higher SQ scores. Conversely, no significant correlation was observed between AQ scores, which reflect overall autistic traits, and EQ scores (6, 8). Therefore, the relationship with hand configurations is likely a feature of systemizing traits, which are a part of ASD-related traits. Our findings suggest that a new measurement of hand configurations, which may be influenced by gene polymorphisms or prenatal sex hormone exposure, can predict some parts of ASD-related traits in the neurotypical population. In particular, the ratio of the distance between the MCP joints of the index finger and the ring finger (*FW_2_3R_* + *FW_3_4R_*) to the length of the middle finger (*FL_3R_*) in the right hand showed a correlation coefficient of >0.4, indicating a considerable degree of correlation. This indicator could be a candidate biomarker for systemizing traits.

As we expected, the present results suggest that unknown gene polymorphisms might affect systemizing traits (i.e., neurodevelopment) and finger development. Previous studies reported that polymorphisms in neurexin were associated with SQ scores (20) and that the expression of the neurexin gene could induce the expression of bone morphogenetic factors during sympathetic neuron development (21). The expression of neurexin possibly influences the developmental process of hand configurations with respect to the distance between the metacarpals. Based on these findings, we could consider that genetic factors may be involved in the development of systemizing traits, which are a part of ASD-related traits. Evolutionary significance may be involved here; for instance, ASD-related traits may have evolutionary advantages when a person concentratedly and repeatedly carries out precise tasks, irrespective of social contexts (58). In particular, systemizing traits are important for systematically understanding and organizing various things, and we speculate that they are indispensable for the construction of modern civilization. The link between these traits and hand configurations may also have evolutionary significance.

Moreover, prenatal sex hormone exposure might relate to the results. As suggested by a previous study, the degree of prenatal androgen exposure could affect the systemizing traits (as measured during routine amniocentesis) (32). A previous study with a larger sample observed that males consistently had much higher systemizing traits than females (56). Furthermore, prenatal androgen exposure promotes bone and cartilage formation, resulting in an increased finger base distance (59). It is known that sex hormones may influence the expression of the *Hox* gene (60–62), which is important for the development of the nervous system and limbs (63, 64). This may lead to variations in hand configurations, including the 2D:4D ratio (65). However, while lower values have been reported for the finger ratios (2D:4D), which have been implicated in prenatal testosterone exposure in individuals with clinically diagnosed ASD, the association between the 2D:4D ratio and the ASD-related traits (AQ, SQ, or EQ scores) in the general population remains uncertain, according to meta-analyses (27). Consistent with previous meta-analyses, no significant correlation between the 2D:4D ratio and SQ score was observed in the present study; nevertheless, a significant correlation (0.35–0.44) between the aspect ratio of the right hand and SQ scores was noted. In addition, as reported in the results, no significant correlation was found between the hand aspect ratio and the 2D:4D ratio in the right hand, suggesting that a different mechanism may be involved. On the other hand, while the aspect ratio of the left hand was not associated with ASD-related traits, there was a significant correlation between the aspect ratio and the 2D:4D ratio in the left hand. It was suggested that there may be different ontogenetic/developmental processes in the left and right hands, but further studies are needed. Furthermore, although a significant sex difference has been reported for the 2D:4D ratio (57), we found no apparent sex differences in the aspect ratio and related hand configurations. Hand size is thought to reflect overall body size and is generally larger in males compared to females (46, 47). We controlled possible effects of sex-related hand size differences by calculating the ratio of the distance between the MCP joints to the length of the middle finger. We speculated that with this manipulation, there would be no apparent sex difference for the indices in the current study. Therefore, we believe that another phenomenon closely related to SQ, apart from prenatal testosterone exposure, is likely to be involved (e.g., gene polymorphisms).

The present study had some limitations. First, the relationship between hand configurations and SQ scores was clearly observed only for the right hand in our sample of right-hand-dominant participants. The possibility exists that it is simply a feature reflecting the frequency of hand use according to hand dominance. For example, an increase in muscle mass due to heavy use of the dominant hand may be primarily involved. We should also note the possible effect of age, as the present study collected data from adolescent to adult participants. However, the effect of age was observed not only in the dominant but also in the nondominant hand. Future studies with left-handed participants and participants of a wider age range, including children, are needed to clarify this. If the correlation between SQ and the aspect ratio found here is due to gene polymorphisms or sex hormone exposure during embryonic development, the correlation should also be observed in children. However, if the frequency of hand use is critical, the correlation should not be observed initially but should appear with development in both left- and right-handed people in the dominant hand. We believe the former is more likely, as the effect of age was also observed in the left hand, where no correlation with SQ was observed. Second, the locations of the MCP joints were visually determined, and the distance was measured from photographs; therefore, these might have contained some errors. In addition, the participants were instructed to place their hands naturally, and some variations in the manner in which the hands were opened and closed were expected to occur. Differences were not expected to significantly affect the measurements of MCP joint distances and finger lengths; however, some degree of error might have occurred. Future studies are required to reveal the relationship between hand configurations using different measurements. For instance, well-controlled direct measurements, such as radiographs, can be used to determine the length and width of the metacarpals and phalanges of the right and left hands (66). It may be necessary to examine the aspect ratio and other metrics and their association with gene polymorphisms. Artificial intelligence (AI)-based analysis may be useful for this purpose. It may be possible to develop novel biomarkers by exploring areas that researchers have previously overlooked. Although not limited to this study, ethical considerations and careful handling are necessary when applying AI analyses. Third, the present study targeted the effects of ASD-related traits in the general population. This study found an association between the aspect ratio of the right hand and the SQ score, which is part of ASD-related traits. Therefore, the findings suggest an association between ASD-related cognitive styles and hand development, but this cognitive style is not necessarily consistent with the diagnosis of ASD. However, it is important to examine the aspect ratio of the hands in individuals with neurodevelopmental disorders, particularly those diagnosed with ASD. Therefore, it may be necessary to investigate participants with a clinical diagnosis of ASD in future studies.

In summary, the current study revealed a correlation between the aspect ratio of the right hand and systemizing traits. Individuals with a greater width between the finger MCP joints (e.g., between the index and ring fingers) relative to the finger length (e.g., the middle finger) of the right hand tended to have higher SQ scores. These results suggest that hand configurations, particularly the width between the finger MCP joints, are related to systemizing traits and that gene polymorphisms or prenatal sex hormone exposure may underlie the relationship.

## Data Availability

The datasets presented in this study can be found in online repositories. The names of the repository/repositories and accession number(s) can be found below: https://osf.io/5xcsj/.
