# Supplementary material for: People with higher systemizing traits have wider right hands
Source: Front Psychiatry. 2024 Sep 5;15:1404559. doi: 10.3389/fpsyt.2024.1404559 (PMC11411187; doi:10.3389/fpsyt.2024.1404559)
Supplement: Supplementary file 1 [file DataSheet1.docx]

Supplementary materials

Supplementary Table 1. Pearson’s correlation coefficients between aspect ratio of hands and ASD-related traits.

|  |  | Right hand | Left hand |
| --- | --- | --- | --- |
| AQ | *r* =  *p =* | -.018  .87 | 0.13  .27 |
| SQ | *r* =  *p =* | **0.33^*^**  **.003** | -0.07  .53 |
| EQ | *r* =  *p =* | -0.019  .86 | -0.17  .12 |
| Age | *r* =  *p =* | **0.30^*^**  **.007** | 0.14  .23 |
| LQ (handedness) | *r* =  *p =* | -0.001  .99 | 0.18  .10 |

Values displayed in bold and asterisks indicate the level of statistical significance (*p < .05/5).

Supplementary Table 2. Pearson’s correlation coefficients between ratios about hand configurations and ASD-related traits.

|  |  | Right hand | | | Left hand | | |
| --- | --- | --- | --- | --- | --- | --- | --- |
|  |  | FW_2_3R_/ FL_3R_ | FW_3_4R_/ FL_3R_ | FW_4_5R_/ FL_3R_ | FW_2_3L_/ FL_3L_ | FW_3_4L_/ FL_3L_ | FW_4_5L_/ FL_3L_ |
| AQ | *r* =  *p =* | -0.007  .95 | -0.024  .83 | -0.005  .97 | 0.082  .47 | -0.086  .44 | -0.045  .69 |
| SQ | *r* =  *p =* | **0.33**^*^  **.002** | **0.34**^*^  **.002** | 0.017  .88 | 0.10  .35 | 0.064  .57 | 0.11  .35 |
| EQ | *r* =  *p =* | -0.014  .90 | -0.035  .76 | 0.012  .92 | -0.036  .75 | .0.15  .19 | -0.025  .82 |
| Age | *r* =  *p =* | **0.31**^*^  **.005** | 0.12  .30 | 0.19  .094 | **0.36^*^**  **< .001** | **0.31^*^**  **.005** | 0.11  .32 |
| LQ  (handedness) | *r* =  *p =* | 0.089  .43 | -0.12  .27 | 0.004  .98 | 0.16  .15 | -0.060  .59 | 0.13  .26 |

Values displayed in bold and asterisks indicate the level of statistical significance (*p < .05 / 15).

Supplementary Table 3. Sex differences in ASD-related traits, laterality quotient (LQ), and ratios about hand configurations.

| A. ASD-related traits and LQ | | | | | | | | | | |
| --- | --- | --- | --- | --- | --- | --- | --- | --- | --- | --- |
|  | | *t* | | *df* | | *p* | | Cohen's *d* | |  |
| AQ |  | -0.93 |  | 66.6 |  | .36 |  | -0.21 |  |  |
| **SQ** |  | **-5.10** |  | **64.6** |  | **< .001** |  | **-1.16** |  |  |
| EQ |  | 0.96 |  | 69.6 |  | .34 |  | 0.22 |  |  |
| LQ |  | 0.59 |  | 70.3 |  | .56 |  | 0.13 |  |  |
|  | | | | | | | | | | |
| Note.  Welch's t-test. | | | | | | | | | | |

| A. Right hand | | | | | | | | | |
| --- | --- | --- | --- | --- | --- | --- | --- | --- | --- |
|  | | *t* | | *df* | | *p* | | Cohen's *d* | |
| *FW_2_3R_/FL_3R_* |  | -0.34 |  | 78.9 |  | .74 |  | -0.074 |  |
| ***FW_3_4R_/FL_3R_*** |  | **-2.68** |  | **74.1** |  | **.009^*^** |  | **-0.60** |  |
| *FW_4_5R_/FL_3R_* |  | -0.29 |  | 75.1 |  | .77 |  | -0.065 |  |
| (*FW_2_3R_* +*FW_3_4R_*)*/FL_3R_* |  | -1.60 |  | 78.6 |  | .11 |  | -0.35 |  |
| (*FW_2_3R_* +*FW_3_4R_* +*FW_4_5R_*)*/FL_3R_* (Aspect ratio) |  | -1.39 |  | 79.6 |  | .17 |  | -0.30 |  |
|  | | | | | | | | | |
| Note.  Welch's t-test. | | | | | | | | | |

| B. Left hand | | | | | | | | | |
| --- | --- | --- | --- | --- | --- | --- | --- | --- | --- |
|  | | *t* | | *df* | | *p* | | Cohen's *d* | |
| *FW_2_3L_/FL_3L_* |  | 1.06 |  | 80.0 |  | .29 |  | 0.23 |  |
| *FW_3_4L_/FL_3L_* |  | 1.60 |  | 79.9 |  | .11 |  | 0.35 |  |
| *FW_4_5L_/FL_3L_* |  | 0.36 |  | 79.8 |  | .72 |  | 0.079 |  |
| (*FW_2_3L_* +*FW_3_4L_*)*/FL_3L_* |  | 0.34 |  | 79.5 |  | .73 |  | 0.075 |  |
| (*FW_2_3L_* +*FW_3_4L_* +*FW_4_5L_*)*/FL_3L_* (Aspect ratio) |  | 0.007 |  | 74.9 |  | .99 |  | 0.002 |  |
|  | | | | | | | | | |
| Note.  Welch's t-test. | | | | | | | | | |

Supplementary Table 4. Hierarchical multiple linear regression analyses for the aspect ratios.

| A. Right hand | | | | | | | | | | | | | |
| --- | --- | --- | --- | --- | --- | --- | --- | --- | --- | --- | --- | --- | --- |
| Model | | Adjusted *R²* | | *R²* Change | | *F* Change | | *df1* | | *df2* | | *p* | |
| First step |  | 0.009 |  | 0.021 |  | 1.75 |  | 1 |  | 80 |  | .19 |  |
| **Second step** |  | **0.15** |  | **0.19** |  | **4.43** |  | **4** |  | **76** |  | **.003** |  |

| Model | | Variables | | *β* | | *t* | | *p* | | *VIF* | |
| --- | --- | --- | --- | --- | --- | --- | --- | --- | --- | --- | --- |
| First step |  | (Intercept) |  |  |  | -61.0 |  | < .001 |  |  |  |
|  |  | Sex |  |  |  | 1.32 |  | .19 |  | 1.00 |  |
| Second step |  | (Intercept) |  |  |  | -15.0 |  | < .001 |  |  |  |
|  |  | Sex |  |  |  | 0.76 |  | .45 |  | 1.56 |  |
|  |  | **Age** |  | **0.32** |  | **2.91** |  | **.005** |  | **1.16** |  |
|  |  | AQ |  | -0.068 |  | -0.57 |  | .57 |  | 1.38 |  |
|  |  | **SQ** |  | **0.29** |  | **2.35** |  | **.021** |  | **1.48** |  |
|  |  | EQ |  | -0.032 |  | -0.27 |  | .79 |  | 1.33 |  |
|  | | | | | | | | | | | |

| B. Left hand | | | | | | | | | | | | | |
| --- | --- | --- | --- | --- | --- | --- | --- | --- | --- | --- | --- | --- | --- |
| Model | | Adjusted *R²* | | *R²* Change | | *F* Change | | *df1* | | *df2* | | *p* | |
| First step |  | -0.012 |  | 0.00 |  | < 0.0001 |  | 1 |  | 80 |  | .99 |  |
| Second step |  | 0.004 |  | 0.066 |  | 1.33 |  | 4 |  | 76 |  | .27 |  |

| Model | | Variables | | *β* | | *t* | | *p* | | *VIF* | |
| --- | --- | --- | --- | --- | --- | --- | --- | --- | --- | --- | --- |
| First step |  | (Intercept) |  |  |  | -30.0 |  | < .001 |  |  |  |
|  |  | Sex |  |  |  | -0.007 |  | .99 |  | 1.00 |  |
| Second step |  | (Intercept) |  |  |  | -6.65 |  | < .001 |  |  |  |
|  |  | Sex |  |  |  | 0.71 |  | .48 |  | 1.56 |  |
|  |  | Age |  | 0.17 |  | 1.53 |  | .15 |  | 1.16 |  |
|  |  | AQ |  | 0.11 |  | 0.86 |  | .39 |  | 1.38 |  |
|  |  | SQ |  | -0.14 |  | -1.06 |  | .30 |  | 1.48 |  |
|  |  | EQ |  | -0.11 |  | -0.83 |  | .41 |  | 1.33 |  |
|  | | | | | | | | | | | |

Supplementary Table 5. Hierarchical multiple linear regression analyses for the ratios.

| A. *FW_2_3R_/FL_3R_* | | | | | | | | | | | | | |
| --- | --- | --- | --- | --- | --- | --- | --- | --- | --- | --- | --- | --- | --- |
| Model | | Adjusted *R²* | | *R²* Change | | *F* Change | | *df1* | | *df2* | | *p* | |
| First step |  | -0.011 |  | 0.001 |  | 0.10 |  | 1 |  | 80 |  | .75 |  |
| **Second step** |  | **0.21** |  | **0.21** |  | **5.13** |  | **4** |  | **76** |  | **.001** |  |

| Model | | Variables | | *β* | | *t* | | *p* | | *VIF* | |
| --- | --- | --- | --- | --- | --- | --- | --- | --- | --- | --- | --- |
| First step |  | (Intercept) |  |  |  | -113.7 |  | < .001 |  |  |  |
|  |  | Sex |  |  |  | 0.32 |  | 075 |  | 1.00 |  |
| Second step |  | (Intercept) |  |  |  | -26.7 |  | < .001 |  |  |  |
|  |  | Sex |  |  |  | -0.56 |  | .58 |  | 1.56 |  |
|  |  | **Age** |  | **0.29** |  | **2.60** |  | **.011** |  | **1.16** |  |
|  |  | AQ |  | -0.068 |  | -0.57 |  | .58 |  | 1.38 |  |
|  |  | **SQ** |  | **0.38** |  | **3.09** |  | **.003** |  | **1.48** |  |
|  |  | EQ |  | -0.045 |  | -0.38 |  | .70 |  | 1.33 |  |
|  | | | | | | | | | | | |

| B. *FW_3_4R_/FL_3R_* | | | | | | | | | | | | | |
| --- | --- | --- | --- | --- | --- | --- | --- | --- | --- | --- | --- | --- | --- |
| Model | | Adjusted *R²* | | *R²* Change | | *F* Change | | *df1* | | *df2* | | *p* | |
| **First step** |  | **0.069** |  | **0.081** |  | **7.03** |  | **1** |  | **80** |  | **.010** |  |
| Second step |  | 0.12 |  | 0.089 |  | 2.04 |  | 4 |  | 76 |  | .097 |  |

| Model | | Variables | | *β* | | *t* | | *p* | | *VIF* | |
| --- | --- | --- | --- | --- | --- | --- | --- | --- | --- | --- | --- |
| First step |  | (Intercept) |  |  |  | -157.0 |  | < .001 |  |  |  |
|  |  | **Sex** |  |  |  | **2.65** |  | **.010** |  | **1.00** |  |
| Second step |  | (Intercept) |  |  |  | -32.9 |  | < .001 |  |  |  |
|  |  | Sex |  |  |  | 1.61 |  | .11 |  | 1.56 |  |
|  |  | Age |  | 0.17 |  | 1.48 |  | .14 |  | 1.16 |  |
|  |  | AQ |  | -0.11 |  | -0.87 |  | .39 |  | 1.38 |  |
|  |  | SQ |  | 0.26 |  | 2.01 |  | .048 |  | 1.48 |  |
|  |  | EQ |  | -0.056 |  | -0.47 |  | .64 |  | 1.33 |  |
|  | | | | | | | | | | | |

| C. *FW_4_5R_/FL_3R_* | | | | | | | | | | | | | |
| --- | --- | --- | --- | --- | --- | --- | --- | --- | --- | --- | --- | --- | --- |
| Model | | Adjusted *R²* | | *R²* Change | | *F* Change | | *df1* | | *df2* | | *p* | |
| First step |  | -0.011 |  | 0.001 |  | 0.082 |  | 1 |  | 80 |  | .78 |  |
| Second step |  | -0.017 |  | 0.045 |  | 0.90 |  | 4 |  | 76 |  | .47 |  |

| Model | | Variables | | *β* | | *t* | | *p* | | *VIF* | |
| --- | --- | --- | --- | --- | --- | --- | --- | --- | --- | --- | --- |
| First step |  | (Intercept) |  |  |  | -131.7 |  | < .001 |  |  |  |
|  |  | Sex |  |  |  | 0.29 |  | .78 |  | 1.00 |  |
| Second step |  | (Intercept) |  |  |  | -27.6 |  | < .001 |  |  |  |
|  |  | Sex |  |  |  | 0.90 |  | .37 |  | 1.56 |  |
|  |  | Age |  | 0.23 |  | 1.89 |  | .063 |  | 1.16 |  |
|  |  | AQ |  | 0.039 |  | 0.30 |  | .77 |  | 1.38 |  |
|  |  | SQ |  | -0.053 |  | -0.39 |  | .70 |  | 1.48 |  |
|  |  | EQ |  | 0.047 |  | 0.37 |  | .72 |  | 1.33 |  |
|  | | | | | | | | | | | |

| D. *FW_2_3L_/FL_3L_* | | | | | | | | | | | | | |
| --- | --- | --- | --- | --- | --- | --- | --- | --- | --- | --- | --- | --- | --- |
| Model | | Adjusted *R²* | | *R²* Change | | *F* Change | | *df1* | | *df2* | | *p* | |
| First step |  | 0.015 |  | 0.027 |  | 2.25 |  | 1 |  | 80 |  | .14 |  |
| **Second step** |  | **0.12** |  | **0.15** |  | **3.31** |  | **4** |  | **76** |  | **.015** |  |

| Model | | Variables | | *β* | | *t* | | *p* | | *VIF* | |
| --- | --- | --- | --- | --- | --- | --- | --- | --- | --- | --- | --- |
| First step |  | (Intercept) |  |  |  | -103.0 |  | < .001 |  |  |  |
|  |  | Sex |  |  |  | -1.50 |  | .14 |  | 1.00 |  |
| Second step |  | (Intercept) |  |  |  | -24.1 |  | < .001 |  |  |  |
|  |  | Sex |  |  |  | -1.26 |  | .21 |  | 1.56 |  |
|  |  | **Age** |  | **0.33** |  | **2.90** |  | **.005** |  | **1.16** |  |
|  |  | AQ |  | 0.10 |  | 0.82 |  | .42 |  | 1.38 |  |
|  |  | SQ |  | 0.17 |  | 1.34 |  | .19 |  | 1.48 |  |
|  |  | EQ |  | <0.001 |  | <0.001 |  | .999 |  | 1.33 |  |
|  | | | | | | | | | | | |

| E. *FW_3_4L_/FL_3L_* | | | | | | | | | | | | | |
| --- | --- | --- | --- | --- | --- | --- | --- | --- | --- | --- | --- | --- | --- |
| Model | | Adjusted *R²* | | *R²* Change | | *F* Change | | *df1* | | *df2* | | *p* | |
| First step |  | -0.011 |  | 0.001 |  | 0.12 |  | 1 |  | 80 |  | .73 |  |
| Second step |  | 0.068 |  | 0.12 |  | 2.70 |  | 4 |  | 76 |  | .037 |  |

| Model | | Variables | | *β* | | *t* | | *p* | | *VIF* |  |
| --- | --- | --- | --- | --- | --- | --- | --- | --- | --- | --- | --- |
| First step |  | (Intercept) |  |  |  | -135 |  | < .001 |  |  |  |
|  |  | Sex |  |  |  | -0.35 |  | .73 |  | 1.00 |  |
| Second step |  | (Intercept) |  |  |  | -30.5 |  | < .001 |  |  |  |
|  |  | Sex |  |  |  | 0.43 |  | .67 |  | 1.56 |  |
|  |  | **Age** |  | **0.33** |  | **2.87** |  | **.005** |  | **1.16** |  |
|  |  | AQ |  | 0.011 |  | 0.089 |  | .93 |  | 1.38 |  |
|  |  | SQ |  | 0.037 |  | 0.29 |  | .76 |  | 1.48 |  |
|  |  | EQ |  | 0.16 |  | 1.32 |  | .19 |  | 1.33 |  |
|  | | | | | | | | | | |  |

| F. *FW_4_5L_/FL_3L_* | | | | | | | | | | | | | |
| --- | --- | --- | --- | --- | --- | --- | --- | --- | --- | --- | --- | --- | --- |
| Model | | Adjusted *R²* | | *R²* Change | | *F* Change | | *df1* | | *df2* | | *p* | |
| First step |  | -0.011 |  | 0.001 |  | 0.11 |  | 1 |  | 80 |  | .75 |  |
| Second step |  | -0.027 |  | 0.035 |  | 0.70 |  | 4 |  | 76 |  | .60 |  |

| Model | | Variables | | *β* | | *t* | | *p* | | *VIF* | |
| --- | --- | --- | --- | --- | --- | --- | --- | --- | --- | --- | --- |
| First step |  | (Intercept) |  |  |  | -136 |  | < .001 |  |  |  |
|  |  | Sex |  |  |  | -0.33 |  | .75 |  | 1.00 |  |
| Second step |  | (Intercept) |  |  |  | -27.1 |  | < .001 |  |  |  |
|  |  | Sex |  |  |  | -0.73 |  | .47 |  | 1.56 |  |
|  |  | Age |  | 0.071 |  | 0.58 |  | .56 |  | 1.16 |  |
|  |  | AQ |  | -0.098 |  | -0.75 |  | .45 |  | 1.38 |  |
|  |  | SQ |  | 0.18 |  | 1.30 |  | .20 |  | 1.48 |  |
|  |  | EQ |  | -0.079 |  | -0.61 |  | .55 |  | 1.33 |  |
|  | | | | | | | | | | | |

Supplementary Table 6. Correlations between 2D:4D ratios and ASD-related traits.

| A. Right hand | | | | | | | | | |
| --- | --- | --- | --- | --- | --- | --- | --- | --- | --- |
|  | |  | |  | | Pearson's *r* | | *p* | |
| 2D4D |  | - |  | AQ |  | 0.006 |  | .96 |  |
| 2D4D |  | - |  | SQ |  | -0.19 |  | .089 |  |
| 2D4D |  | - |  | EQ |  | -0.09 |  | .43 |  |
|  | | | | | | | | | |
|  | | | | | | | | | |

| B. Left hand | | | | | | | | | |
| --- | --- | --- | --- | --- | --- | --- | --- | --- | --- |
|  | |  | |  | | Pearson's r | | p | |
| 2D4D |  | - |  | AQ |  | 0.17 |  | .14 |  |
| 2D4D |  | - |  | SQ |  | -0.13 |  | .26 |  |
| 2D4D |  | - |  | EQ |  | -0.056 |  | .62 |  |
|  | | | | | | | | | |
|  | | | | | | | | | |

Supplementary Table 7. Correlations between 2D:4D ratios and ratios about hand configurations.

| A. Right hand | | | | | | | | | |  |
| --- | --- | --- | --- | --- | --- | --- | --- | --- | --- | --- |
|  | |  | |  | | Pearson's *r* | | *p* | |  |
| 2D4D |  | - |  | *FW_2_3L_/FL_3L_* |  | -0.023 |  | .84 |  | |
| 2D4D |  | - |  | *FW_3_4L_/FL_3L_* |  | -0.017 |  | .88 |  | |
| 2D4D |  | - |  | *FW_4_5L_/FL_3L_* |  | -0.064 |  | .57 |  | |
| 2D4D |  | - |  | (*FW_2_3L_* +*FW_3_4L_*)*/FL_3L_* |  | -0.023 |  | .84 |  | |
| 2D4D |  | - |  | (*FW_2_3L_* +*FW_3_4L_* +*FW_4_5L_*)*/FL_3L_* (Aspect ratio) |  | -0.045 |  | .69 |  | |
|  | | | | | | | | | |  |
|  | | | | | | | | | |  |

| B. Left hand | | | | | | | | |  |
| --- | --- | --- | --- | --- | --- | --- | --- | --- | --- |
|  | |  | |  | | Pearson's *r* | | *P* |  |
| 2D4D |  | - |  | *FW_2_3L_/FL_3L_* |  | 0.222 |  | .045 |  |
| 2D4D |  | - |  | *FW_3_4L_/FL_3L_* |  | -0.102 |  | .364 |  |
| 2D4D |  | - |  | *FW_4_5L_/FL_3L_* |  | 0.252 |  | .022 |  |
| 2D4D |  | - |  | (*FW_2_3L_* +*FW_3_4L_*)*/FL_3L_* |  | 0.211 |  | .057 |  |
| 2D4D |  | - |  | (*FW_2_3L_* +*FW_3_4L_* +*FW_4_5L_*)*/FL_3L_* (Aspect ratio) |  | 0.350 | * | .001 |  |
|  | | | | | | | | |  |
| * *p* < .05/5 | | | | | | | | |  |
